# Cryo-EM maps of human DNA polymerase ε should be reevaluated in light of its unexpected behavior in vitro

**DOI:** 10.1073/pnas.2533320123

**Published:** 2026-03-06

**Authors:** Johann J. Roske, Joseph T. P. Yeeles

**Affiliations:** ^a^Medical Research Council, Protein and Nucleic Acid Chemistry Division Division, Laboratory of Molecular Biology, Cambridge CB2 0QH, United Kingdom

DNA polymerase epsilon (Polε) synthesizes the nascent leading strand during chromosome replication. To achieve high fidelity, Polε possesses proofreading activity to recognize and excise misincorporated nucleotides from the nascent DNA strand. Our cryo-EM structures of human Polε-PCNA bound to (mismatch-containing) DNA substrates revealed Polε in states poised for strand extension, mismatch excision, and in intermediate conformations that delineate a switching mechanism between polymerase and exonuclease activities (1). Wang et al. also performed cryo-EM analysis on Polε-PCNA with a DNA scaffold containing a 3′-terminal mismatch in the nascent strand (2). However, they only reported a “blocked conformation” where the DNA sits in the center of the complex without binding in either of the active sites and concluded that a preformed mismatch “traps” Polε-PCNA, rendering it unable to perform proofreading.

Using similar DNA scaffolds that contained a 5′ single-stranded overhang on one side (no mismatch) (Fig. 1*A*), we repeatedly observed that human Polε-PCNA assembles not only at the 3′-junction as intended (Fig. 1*B*) but also on the blunt-ended face of the DNA scaffold (Fig. 1 *C* and *D*) (1). Using streptavidin to mark the biotinylated blunt end, we confirmed this observation (Fig. 1*E*) and dismissed it as artifactual because blunt-end engagement does not represent the active mode of Polε (1).

**Fig. 1.**
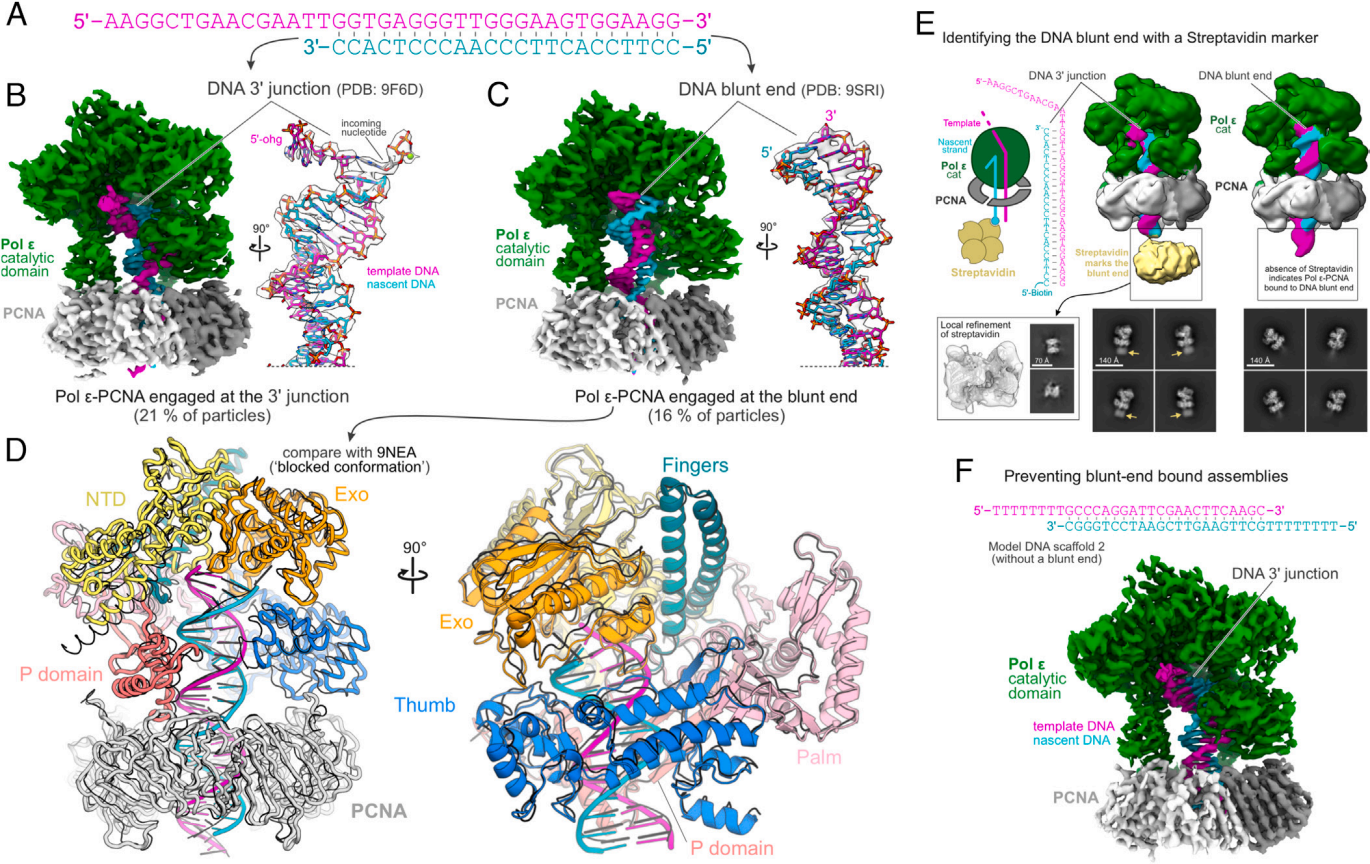
(*A*) DNA scaffold with a single 3′-junction and a blunt end. (*B* and *C*) Cryo-EM maps of Polε-PCNA bound to either the 3′ junction (*B*) or the blunt-ended face (*C*) of the DNA scaffold shown in (*A*). Cryo-EM density for the DNA is shown together with the molecular model, respectively. See ref. 1 methods for cryo-EM data processing pipeline. (*D*) Ribbon representation of Polε-PCNA bound to a DNA blunt end (colored by subdomain, PDB: 9SRI) superimposed with the reported blocked conformation (PDB: 9NEA, shown in black). Models were aligned on the Polε catalytic domain. PCNA is hidden for clarity in the panel on the *Right*. (*E*) Streptavidin marks the blunt end of a DNA scaffold with a biotin modification. The absence of the streptavidin signal indicates that Polε-PCNA is bound to the DNA blunt end. (*F*) Cryo-EM structure of Polε-PCNA (EMD-55724, 2.9 Å resolution) reconstituted on a DNA scaffold that lacks blunt ends.

Strikingly, blunt end-bound Polε-PCNA (PDB: 9SRI, EMD-55141) is virtually identical to the blocked conformation reported in ref. 2 (9NEA, Fig. 1*D*). Therefore, in our view, the blocked conformation likely represents blunt end-bound Polε-PCNA, rather than a response of the complex to a preformed mismatch. Consequently, the data presented in ref. 2 would not support the conclusion that Polε-PCNA is unable to proofread substrates with a preformed mismatch. To circumvent artifactual blunt-end binding by Polε, we propose the use of DNA scaffolds with 5′-overhangs on both sides that only result in reconstructions of Polε-PCNA engaged at a 3′-junction (Fig. 1*F*).

To generate a mismatch “in situ,” Wang et al. incubated exonuclease-deficient Polε (Polε^exo−^) with a primer/template scaffold, PCNA and dTTP, assuming Polε^exo−^ would only introduce a single mismatch (Fig. 2*A*) (2). However, the dTTP concentration used was 500 µM, which is 10 to 20-fold physiological levels (3, 4), and a control experiment examining the outcomes of these reactions is lacking. Because high dNTP concentrations and exonuclease deficiency can increase mismatch incorporation (refs. 5–7 and others), we hypothesized that 500 µM dTTP might cause Polε^exo−^ to produce a range of heterogenous extension products that would have to be considered during interpretation of cryo-EM data. By performing primer extension reactions using the same DNA sequences and dTTP concentrations as in ref. 2, we found that a single mismatch at the nascent terminus is indeed not the only outcome (Fig. 2 *B* and *C*). Polε^exo−^ stalled before generating a mismatch (+3-product) and also incorporated multiple nucleotides beyond the initial mismatch, presumably generating bulged readthrough products. Notably, this behavior was influenced by the template DNA sequence (Fig. 2*D*). These data potentially have important implications for the atomic modeling and conclusions drawn by Wang et al. and illustrate the need for caution when using high dNTP concentrations with exo− DNA polymerases.

**Fig. 2.**
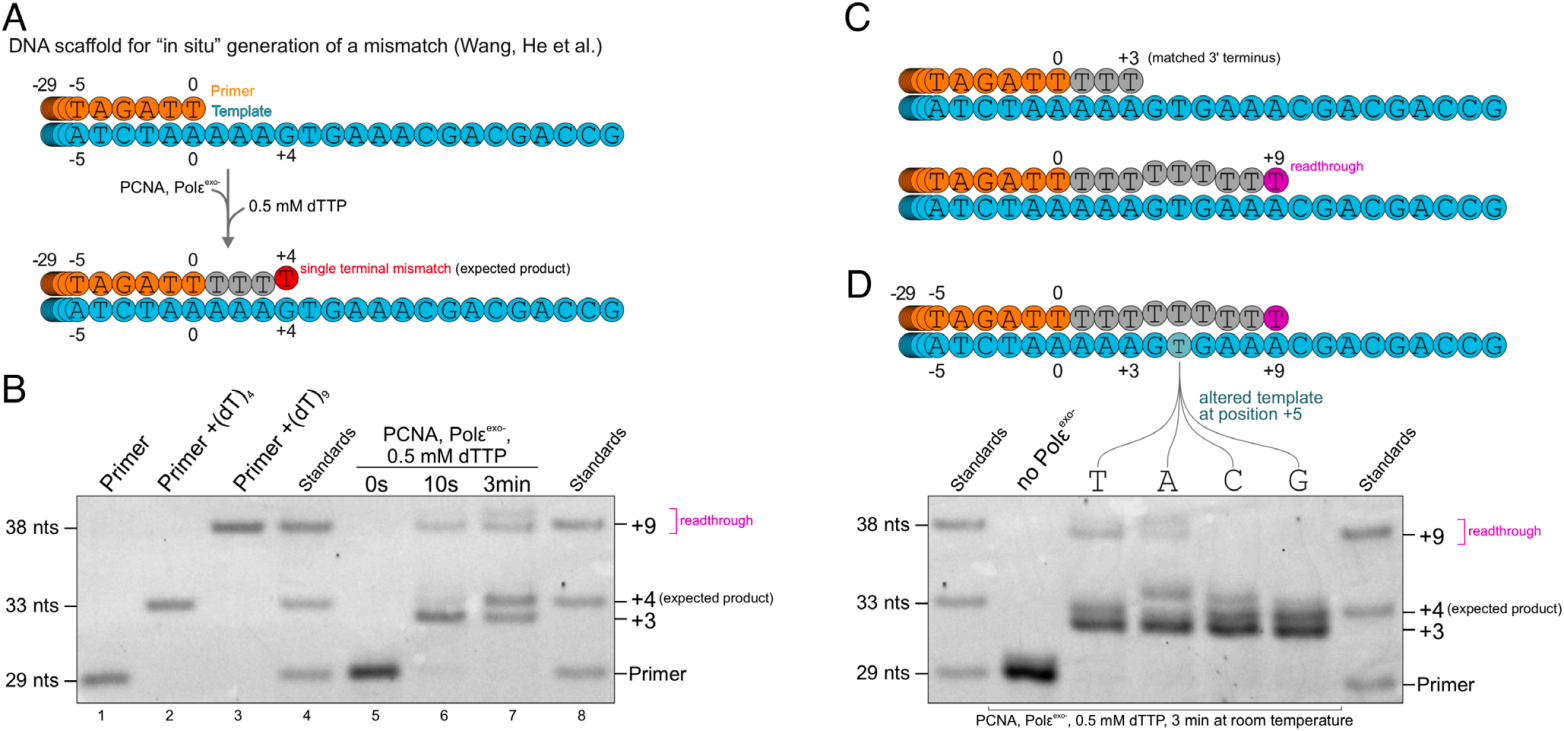
(*A*) Schematic of the primer/template scaffold used by Wang et al. to generate a mismatch by Polε^exo−^-PCNA upon addition of dTTP. The extension product that is assumed for molecular modeling contains four additional dT, forming a single terminal dT·dG mismatch. (*B*) PAGE analysis of primer extension reactions with the same DNA sequences (with additional Cy5-label on the 5′-terminus of the primer for imaging) after 10 s (lane 6) and 3 min (lane 7) at room temperature. Oligodeoxynucleotides representing extension by 0, 4, or 9 dT-nucleobases serve as standards (lanes 1-4 and 8). Observed products are indicated on the right of the gel. (*C*) Schematic of additional products observed in the primer extension reaction. (*D*) Altering the templating base at position +5 from dT to dA/dC/dG, respectively, influences the behavior of Polε^exo−^-PCNA. Reactions were run as in (*B*) for 3 min at room temperature.

## References

[1] J. J. Roske, J. T. P. Yeeles, Structural basis for processive daughter-strand synthesis and proofreading by the human leading-strand DNA polymerase Pol epsilon. Nat. Struct. Mol. Biol. **31**, 1921–1931 (2024). 39112807 10.1038/s41594-024-01370-y PMC11638069

[2] F. Wang, Q. He, M. E. O’Donnell, H. Li, The proofreading mechanism of the human leading-strand DNA polymerase epsilon holoenzyme. Proc. Natl. Acad. Sci. U.S.A. **122**, e2507232122 (2025). 40440070 10.1073/pnas.2507232122 PMC12146725

[3] C. K. Mathews, DNA precursor metabolism and genomic stability. FASEB J. **20**, 1300–1314 (2006). 16816105 10.1096/fj.06-5730rev

[4] C. Y. Huang, M. Yague-Capilla, D. Gonzalez-Pacanowska, Z. F. Chang, Quantitation of deoxynucleoside triphosphates by click reactions. Sci. Rep. **10**, 611 (2020). 31953472 10.1038/s41598-020-57463-3 PMC6969045

[5] K. A. Eckert, T. A. Kunkel, DNA polymerase fidelity and the polymerase chain reaction. PCR Methods Appl. **1**, 17–24 (1991). 1842916 10.1101/gr.1.1.17

[6] L. V. Mendelman, J. Petruska, M. F. Goodman, Base mispair extension kinetics. Comparison of DNA polymerase alpha and reverse transcriptase. J. Biol. Chem. **265**, 2338–2346 (1990). 1688852

[7] S. R. Barbari , Enhanced polymerase activity permits efficient synthesis by cancer-associated DNA polymerase ϵ variants at low dNTP levels. Nucleic Acids Res. **50**, 8023–8040 (2022). 35822874 10.1093/nar/gkac602 PMC9371911

